# Smartphone-Based Tracking of Sleep in Depression, Anxiety, and Psychotic Disorders

**DOI:** 10.1007/s11920-019-1043-y

**Published:** 2019-06-04

**Authors:** Talayeh Aledavood, John Torous, Ana Maria Triana Hoyos, John A. Naslund, Jukka-Pekka Onnela, Matcheri Keshavan

**Affiliations:** 1 Department of Psychiatry, University of Helsinki, P.O. Box 22, Välskärinkatu 12 A, FI-00014 Helsinki, Finland; 2 Department of Computer Science, Aalto University, Espoo, Finland; 3 Division of Digital Psychiatry Beth Israel Deaconess Medical Center, Harvard Medical School, Boston, MA USA; 4 Department of Psychiatry, Beth Israel Deaconess Medical Center, Harvard Medical School, Boston, MA USA; 5 Department of Global Health and Social Medicine, Harvard Medical School, Boston, MA USA; 6 Department of Biostatistics, Harvard T.H. Chan School of Public Health, Boston, MA USA

**Keywords:** Sleep, Psychotic disorders, Mental illness, Circadian rhythms, Smartphones, Wearable

## Abstract

**Purpose of Review:**

Sleep is an important feature in mental illness. Smartphones can be used to assess and monitor sleep, yet there is little prior application of this approach in depressive, anxiety, or psychotic disorders. We review uses of smartphones and wearable devices for sleep research in patients with these conditions.

**Recent Findings:**

To date, most studies consist of pilot evaluations demonstrating feasibility and acceptability of monitoring sleep using smartphones and wearable devices among individuals with psychiatric disorders. Promising findings show early associations between behaviors and sleep parameters and agreement between clinic-based assessments, active smartphone data capture, and passively collected data. Few studies report improvement in sleep or mental health outcomes.

**Summary:**

Success of smartphone-based sleep assessments and interventions requires emphasis on promoting long-term adherence, exploring possibilities of adaptive and personalized systems to predict risk/relapse, and determining impact of sleep monitoring on improving patients’ quality of life and clinically meaningful outcomes.

## Introduction

The rapid consumer adoption of smartphones and wearables has increased both the interest in and the feasibility of mobile health across many areas of medicine. In psychiatry, there has been an increased interest in these technologies reflected by both growing research efforts in this area and numerous consumer apps targeting mental health conditions [1•]. While clinical evidence for the effectiveness of many of these apps to aid in the diagnosis, monitoring, and adjunctive treatment of psychiatric conditions is limited and lacking at present, there has been even less focus on the ability of smartphone apps to track and promote positive health behaviors such as sleep among persons living with mental illness. Given the robust literature documenting the important link between sleep and mental health, there is potential for emerging digital technologies to concurrently support the monitoring and tracking of mental health and sleep with the aim of achieving improved health and well-being for individuals living with mental illness.

In this review, we explore the current evidence on the use of smartphones and wearables for tracking and monitoring sleep in persons living with mental illness. Specifically, we seek to summarize the state of the science as it relates to digital interventions for sleep and mental health and consider future research directions. We also discuss the current shortcomings of the literature and propose solutions for using smartphone apps and wearables to monitor sleep in patients with psychiatric disorders.

## Sleep and Psychiatric Disorders

Across all psychiatric illnesses, sleep is often both an important clinical symptom and an important therapeutic target [2]. Sleep disturbances are also associated with lower quality of life among patients [3]. Its importance is underscored by the finding that sleep abnormalities in patients with psychiatric diagnoses are associated with increased risk of suicide [4, 5]. Sleep disturbances are usually a marker for diagnosis of mood disorders [4], and they are a risk factor for developing anxiety and depression [6]. Moreover, insomnia can influence the trajectory of depression, affecting the severity, duration, and relapse rates of the disorder [7]. In psychotic disorders, sleep disturbances are commonly associated with the prodromal phase [8] and may be an important early marker of the premorbid phase of the disease [9]. Sleep disturbances are also often one of the earliest signs of relapse and decompensation in psychotic disorders [10]. In addition, obstructive sleep apnea has a high prevalence in mood and psychotic disorders [11, 12] but is often undetected.

Patients with major depressive disorder and schizophrenia are known to have some common sleep architecture abnormalities including increased sleep latency, decreased total sleep time, and decreased sleep efficiency index [13–15]; furthermore, patients with these diagnoses have lower amplitude slow wave activity (SWA) compared to healthy controls [14]. In addition, positive and negative symptoms for schizophrenia have been correlated with the aforementioned abnormalities. Likewise, sleep abnormalities have been associated with bipolar disorder, including reduction of total sleep time in the mania phase and increase of total sleep time in the depressive phase, decreased rapid eye movement (REM) latency, and increased REM density [16]. Recent research on sleep in psychotic disorders has also produced new data on potential mechanisms of illness. Studies have found that sleep spindles, a feature of stage two non-REM sleep, which may facilitate synaptic plasticity and memory consolidation, are reduced in patients with schizophrenia [17, 18, 19]. Sleep spindle abnormalities are also associated with bipolar disorder [20] and major depressive disorder [21].

## Technology for Detecting Sleep Abnormalities: Existing Evidence

Technology has proven valuable for supplementing the assessment of sleep patterns and abnormalities in clinical settings. Polysomnography (PSG), which involves recording the biphysiological characteristics of sleep including brain waves, eye movements, muscle activity, and heart rhythm during sleep, is considered a gold standard for detecting sleep abnormalities in many psychiatric disorders [22••]. However, PSG is not widely available or convenient for the majority of patients. In clinical practice, sleep disturbances are often assessed via clinical interviews. Commonly, this assessment is based on self-reported sleep measurements, which are susceptible to recall bias and inaccuracies [23] [24]. A good example of this phenomenon is given by Dagan et al. [25] where the researchers found that post-traumatic stress disorder (PTSD) patients tended to misperceive sleep problems and found no correlation between patients’ self-reports and actigraphy measurements for sleep duration, sleep efficiency, activity, and number of sleep/wake transitions. Given the cognitive impairment associated with psychotic disorders, and the false beliefs linked to depressive and anxiety disorders, it is likely that self-reported sleep symptoms may not be accurate.

Actigraphy has emerged as a more practical method for recording patients’ circadian rhythms [26]. Actigraphs are compact devices that allow tracking of sleep/wake patterns for long periods of time in patient’s own environment, but their downside is that they cannot measure sleep staging. Despite this limitation, they are used in the clinical evaluation of treatment benefits for some sleep disorders [27, 28]. Like electroencephalogram (EEG) and PSG, actigraphs have been extensively studied [25, 27–34]. Compared to PSG, sleep parameters measured using actigraphy can be highly correlated in major depressive disorder and anxiety disorders [35–37], as well as both bipolar disorder and schizophrenia spectrum disorders [30].

## Smartphones, Wearables, and New Opportunities for Sleep Monitoring and Intervention

Recent advances in wearables and mobile applications have created new opportunities for symptom tracking and intervention in psychiatry and for assessment of sleep patterns [38••, 39]. Prior technologies such as PSG or actigraphy are not part of an individual’s daily routine, whereas smartphones are ubiquitous [40] and can yield new opportunities to track behaviors in a non-invasive manner. Moreover, smartphones have embedded sensors that can facilitate data acquisition (e.g., accelerometer, microphone), offering novel opportunities to passively monitor patients in their natural environments. In recent years, studies have increasingly utilized smartphones for measuring individuals’ behavior in naturalistic settings. Several of these studies have focused on measuring different features of sleep, such as sleep onset and wake-up time [41•], sleep midpoint and duration [42], disruptions [43], chronotype [44•], and activity rhythms [45] in the general population as well as patients with psychiatric disorders [46••, 47].

In addition to sleep assessment and monitoring, today’s smartphones have the potential to provide sleep related interventions, such as insomnia cognitive behavioral therapy (CBTi), and they can translate data into immediately actionable adjunctive treatments. Thus in mood, anxiety, and psychotic illnesses, where sleep disorders are often outcome predictors, highly prevalent, and associated with increased morbidity and mortality—smartphones offer promising, low-cost, accessible, and practical tools to assist with the diagnosis, monitoring, and in some cases treatment [23, 33, 48–52]. In the sections that follow, we summarize examples identified from the published literature on the use of smartphones to measure sleep parameters and interventions in patients with depression, anxiety, and psychotic disorders.

In this report, we searched PubMed, Embase, PsycINFO, PsycARTICLES, and Web of Science to identify recent studies using smartphones for assessment of sleep among persons with mental illness. Specifically, we searched for original, peer-reviewed, research articles published in English covering three broad concepts: (1) sleep (e.g. “insomnia,” “circadian rhythm”); (2) mobile sensing technology (e.g., “smartphone,” “app,” “wearable,” “sensor”); and (3) mood, anxiety, and psychotic disorders (e.g., “depression,” “bipolar disorder,” “schizophrenia”). We screened around 5000 articles and considered only reports of studies that reported explicitly to have measured sleep-related outcomes with smartphones among patients with psychiatric disorders (e.g., major depression disorder, anxiety disorder, and psychotic disorders including schizophrenia, schizoaffective, and bipolar disorders). We did not consider any papers featuring case reports, protocols, literature reviews, or editorials. We realize that as our process was not systematic, there are some papers we may not have reviewed or included here.

## Evidence for Using Smartphones for Sleep Monitoring and Intervention Among Patients with Psychiatric Disorders

With our limited search strategy, we found eight interesting examples using smartphones and wearable technology for sleep tracking, monitoring, or intervention among individuals with psychiatric disorders. Among them, six studies used different smartphone applications for monitoring sleep and delivering targeted instructional content related to sleep hygiene, and two studies included the use of wearable devices for tracking sleep patterns. First, Schaffer et al. [53] performed a 6-week open-label clinical trial to analyze the effect of quetiapine XR for depressed patients who had not reported improvement with an antidepressant treatment. In this study, smartphones were used to gather daily self-reported data from 26 individuals by administering electronic versions of the 16-item Quick Inventory of Depressive Symptoms-Self Report (QIDS-SR) and 7-item Hospital Anxiety and Depression Scale-Anxiety subscale (HADS-A). In addition, clinical assessments were performed during weeks 1, 2, 4, and 6. The authors found very early changes in the depressive and anxiety symptoms of the subjects reported via smartphones. The greatest change in depression symptoms was detected in the sleep item, although it only accounted for less than one third of the overall change.

Second, in a study by Kane et al. [54], wearable chest sensors were used for monitoring sleep characteristics in a group of 28 adults with bipolar disorder (*n* = 12) and schizophrenia (*n* = 16) over a 4-week period. The sensors, which were integrated with smartphones, were part of the digital health feedback system, a networked system that acquired, summarized, communicated, and displayed information about medication use (ingestion), health status, and daily activities. As part of this study, sleep duration and disruption were derived from the accelerometer data collected by the wearable sensor, while the sleep quality was self-assessed on a Likert scale and entered into a smartphone app. Interestingly, there were no distinguishable differences in sleep duration or disruption between bipolar and schizophrenia patients. Spearman rank correlation coefficients, between the system-derived and the self-reported sleep metrics, were 0.3 for duration and 0.2 for sleep disruption. The authors point out that these correlations are not meaningful and highlight the well-known differences between objective and subjective measurements of sleep in patients with depression and schizophrenia as a possible reason for the finding. The authors also suggest that smartphones and sensors are feasible and useful for monitoring changes in sleep for psychotic patients.

Third, Ben-Zeev et al. [55] employed smartphones to support psychiatric illness self-management among patients with schizophrenia, and sleep was included as one of the targets. The app, called FOCUS, was piloted with 33 patients over a 1-month period and in part provided on-demand psychoeducation on sleep. There were no significant changes in patients’ beliefs about sleep difficulties or medication before and after the intervention. This outcome may be explained by the fact that the sleep intervention component of FOCUS was not designed to provoke short-term relief but rather to promote long-term behavior changes and management of psychiatric symptoms, for example, by modifying sleep hygiene. Thus, it is possible that the trial period was too short to capture changes in sleep. In a later proof-of-concept study, Ben-Zeev et al. utilized FOCUS-Audio/Video (FOCUS-AV) [56], another version of the FOCUS application, which has all the modules of FOCUS in video format. In this study, completed over a month by nine out of ten original participants all with schizophrenia spectrum disorder, participants had access to videos. In these videos, a clinician offered shortened versions of management strategies that are normally administered during a live in-person therapy session. Patients could access these videos on demand. On average, patients accessed the sleep module 8.8 times throughout the study (third rank among all modules), suggesting high interest in improving sleep hygiene among this group of patients. Based on the feedback from the patients, the authors describe these video-based interventions as usable and understandable as well as highly engaging.

Fourth, MoodRhythm is a smartphone application designed for sleep and disturbance of rhythms in affective disorders [57]. In a 4-week long study [58], seven patients with bipolar disorder were given loaner smartphones with MoodRhythm preinstalled. Social rhythms therapy is a method used for treatment of patients with bipolar disorder in which the stability of rhythms of daily life events is assessed by social rhythm metric (SRM) by self-report (pen and paper). The MoodRhythm app uses machine learning to estimate SRM scores from passively collected smartphone data. Among the five social activities that SRM measures, one of them is “out of bed” and “to bed” times. The users’ input (SRM score) serves as the ground truth in this model. Using this approach, the authors inferred subjects’ scores and managed to distinguish between stable rhythm days (SRM ≥ 3.5) and unstable rhythm days (SRM < 3.3) with good accuracy (precision 0.85 and recall 0.86).

Fifth, Faurholt-Jepsen et al. [51] report on a 6-month clinical trial with 61 bipolar patients using MONitoring, treAtment and pRediCtion of bipolAr disorder episodes MONARCA, a smartphone-based self-monitoring system, compared to a control group without MONARCA. No statistically significant correlation was found between self-reported sleep duration and the Hamilton Depression Rating Scale (HDRS-17) scores, although the authors report a negative correlation between self-report and Young Mania Rating Scale (YMRS) scores. A previous study on MONARCA [59•] collected self-reported sleep duration but reports no results on it.

Sixth, Ben-Zeev et al. [60] and Wang et al. [61•] describe a study with a symptom prediction system called CrossCheck, which tracks schizophrenia symptoms based on Brief Psychiatric Rating Scale (BPRS) in 36 recently discharged outpatients with schizophrenia over a period of 2 to 12 months. The CrossCheck symptom prediction system predicts the BPRS score every week based on passive smartphone data and ecological momentary assessment (EMA) collected over 30 days. In clinic visits, which varied in frequency from weekly to monthly, the clinicians administered a seven-item BPRS. The outcome of the BPRS scores later served as the ground truth when predicting BPRS scores collected passively using the smartphone. This study is not directly about sleep; however, different sleep parameters are inferred from passive data (duration, bed time, rise time) also one of the EMA questions is “have you been sleeping well?” which is asked three times a week. Seventh, in an 8-week pilot study with 15 patients with schizophrenia (14 completed with varying level of adherence) conducted by Meyer et al. [62], the aim was to use consumer wearable devices for early detection of relapse by monitoring disturbances in the sleep–wake cycle. In this pilot study, each participant was given an exercise tracker (Fitbit Charge HR) and a loaner smartphone with the Purple Robot and SleepSight apps preinstalled. Study participants were instructed to submit sleep diaries and answer questions about the severity of their symptoms through SleepSight on a daily basis. These entries served as ground truth for the passively collected data. In this pilot study, feasibility was explored based on participants’ adherence to the technology and wearable devices, as well as the agreement between subjective and objective measurements of sleep. Lower sleep diary completion and symptom diary completion rates were associated with negative symptoms (spearman correlation with sleep diary completion *ρ* = − .49, *P* < .05 and symptom diary completion *ρ* = − .40, *P* < .01). Thirteen out of 14 participants met the criteria for 70% feasibility threshold for completion of the daily sleep diary on SleepSight app, with a mean average of 91% of all questionnaires completed. In addition, 12 out of 14 participants met the feasibility criteria for completion of the symptom diary, with a mean average of 88% of filling out the questionnaires. The authors concluded that patients with schizophrenia show interest in sleep disturbances as an indicator of relapse and that they appear willing to use consumer wearable devices and technologies for sleep monitoring. However, lower adherence with active monitoring towards the end of the study suggests that passive monitoring may be more acceptable for patients over longer duration.

Finally, a study by Staples et al. [46••] focuses on sleep and explores use of smartphone data for monitoring sleep among patients with schizophrenia. In this study, 17 patients currently in treatment for schizophrenia installed the Beiwe app on their phones, which collected passive data continuously and EMA data three times a week. The subjects were administered the Pittsburgh Sleep Questionnaire Inventory (PSQI) in clinic monthly, and the PSQI scores were then compared to the EMA and sleep assessments based on the passive data (here, accelerometer data) gathered from the app. In classifying sleep quality either as low or high, the authors report 85% agreement between in-clinic PSQI scores and EMA data. Estimates of sleep duration based on smartphone accelerometer data were moderately correlated (*r* = 0.69, 95% CI 0.23–0.90) with EMA. By combining EMA data and accelerometer data, the authors predicted PSQI scores for all subjects with high accuracy (mean average error = 0.75). Even though missing data emerged as a concern in this study, the authors conclude that smartphone monitoring of sleep in individuals with schizophrenia could possibly be used for predicting in-clinic sleep metrics. In fact, it is expected that missingness is generally a concern with passively collected smartphone data; most of the reviewed papers may have simply ignored this problem as its reliable detection requires collection and analysis of raw sensor data, as was done in this final paper.

## Future Possibilities and Pitfalls

While we identified relatively few studies using smartphone technology to monitor sleep in mood, anxiety, and psychotic disorders, we expect that this topic will continue to gain greater research interest as smartphones and other consumer devices become more ubiquitous. This is evident from the growing number of publications in this area in the past couple of years. Despite the small number of studies, we can draw several important insights from this literature. First, most of the studies suggest that smartphones are suitable for tracking patients’ activity and could be used to find new biomarkers of mental disorders, and some studies conclude that it is feasible to monitor changes in sleep characteristics using wearable sensors [46••, 54, 55, 62]. Second, smartphones are a potential tool for collecting both passive and active data from subjects. Passive data can be collected by means of smartphones’ embedded sensors, while active data can be obtained by using a mobile-friendly survey. Third, because most of the sensors used for sleep monitoring are already included in smartphones, it may not be necessary to deploy separate wearables for gathering information solely about sleep. For example, recent studies suggest that smartphones can be adapted and used to measure and classify populations with sleep problems [63–65]. Importantly, we did not encounter any studies that sought to replicate or reproduce research results using consumer mobile technologies for sleep or any studies that reported improvement in clinically meaningful sleep and mental health outcomes over time, raising the need for further research.

There are also several limitations regarding what smartphone data can tell us about sleep that require careful consideration. For example, currently available consumer mobile devices like smartphones and smartwatches are not equipped with medical grade sensors. Additionally, sleep staging is not feasible at present with smartphone data alone. Nevertheless, the ability of smartphones to track social and behavioral markers offers a unique opportunity to better understand the complexity of mental disorders. Other valuable data, such as duration and hours of sleep, can already be collected; as the methods for inferring these and other sleep parameters improve, this approach is expected to provide important insights into psychiatric illnesses. Overall, smartphones can be helpful for collecting rich human behavioral data with minimal cost if patients can use their own devices [66]. Therefore, tracking and monitoring sleep outcomes may integrate seamlessly into the daily lives of individuals living with psychiatric disorders, offering potential to yield insights about the important relationship between sleep quality and sleep patterns and mental health symptoms.

While current use of smartphones in this context involves measuring sleep via self-reported surveys, new methods and algorithms can be developed to measure sleep using passive data, reducing the need for active user input in data collection. Questionnaires like the PSQI can be employed to learn about subjects’ behavioral patterns and can contribute to training algorithms for extracting information from passive data. In addition, simple logical rules and decision trees can be implemented to infer sleep/wake status based on passive data, and Bayesian methods can be applied to encode prior beliefs and increase the robustness of predictions and inference [67]. Based on these observations, it seems clear that smartphones have substantially more potential to support the monitoring of sleep related problems than what is currently reflected in the literature. At this time, it seems appropriate that consumer wearable devices like smartphones and smartwatches could augment sleep monitoring. Furthermore, increased emphasis is needed on the potential utility of smartphone technologies for delivering treatment interventions beyond active or passive symptom monitoring. For instance, digital prompts could be tailored to patients’ needs by leveraging real time insights collected through sleep monitoring applications in order to facilitate treatment of sleep problems and improve mental health outcomes.

External sensors are one possibility to enhance the existing capabilities of smartphones [65] as they can be attached to the devices, potentially improving the accuracy and reliability of data capture. For example, smartphone apps have been used to identify patients with obstructive sleep apnea, a disorder that is common in patients with mood and psychotic disorders [64, 68, 69]. However, wearable sensors suffer from even lower adherence and compliance than smartphone applications, and thus, there appears to be a compromise between study adherence and data that must be evaluated. Although the studies summarized here reported fairly high average adherence, the duration of the studies was relatively short, with all being 6 months or less. Because of the nature of many psychiatric illnesses, it is unclear what long-term adherence can be expected, especially during severe phases of mania and/or depression.

In psychiatric disorders, sleep staging including quantification of slow wave sleep and spindles can have potential diagnostic and prognostic applications [15, 18]. Changes in sleep are also early warning signs of relapse in schizophrenia or conversion in schizophrenia prodrome. Technology is now becoming available to use portable EEG electrodes to enable this monitoring via smartphones [70] and other portable devices [71]. While further work is needed to examine feasibility and utility of these applications, especially regarding long-term use, their potential is promising.

As surveys captured using smartphone technologies are a valid form of data collection [65, 72], it is important to compare smartphone-based sleep surveys with objective measurement of sleep disturbances in mental disorder patients. Since PSG is the gold standard in sleep research, it is also necessary to examine how smartphone sensors compare to PSG in order to understand the limitations of smartphone-based measurements. For example, one recent study from the general population compared results collected from an app versus PSG measurements, finding that the app performed poorly [73]. However, these results do not generalize to other smartphone apps, and given the wide variation in quality and technical features, the performance of each app may need to be evaluated individually. Kolla et al. [74••] reported on studies that compared smartphones with PSG/actigraphy, but since most of these studies were carried out in healthy individuals, it is difficult to generalize those results to clinical populations.

Another potential use of smartphone technology is intervention for treatment of sleep problems. By using smartphones, interventions could be tailored to individuals’ specific symptoms at specific times, motivating patients to use preventive strategies while coping with real life situations and critical moments. This alone could lead to a reduction of the intensity of the clinic-based sections and improve the patients’ quality of life significantly [50]. Despite FOCUS yielding promising results in the intervention field, interventions related to improving sleep hygiene did not have significant changes in the subjects’ beliefs; Ben-Zeev et al. suggest that this might have been because of the short duration of the trial [55] leaving the topic open for further investigation. However, since patients’ participation in active monitoring may decrease drastically over time, future studies should also aim to find the right balance between the amount of active and passive monitoring and to find ways to encourage patients to increase their adherence to active participation.

Smartphones can also directly deliver sleep interventions and there is a rapidly emerging evidence for delivering CBT insomnia [75]. Randomized trials suggest benefit in those with self-reported symptoms of insomnia [76••] and evidence for this modality for psychotic disorders will likely soon emerge.

## Conclusions

We reviewed the use of smartphones in sleep research in mood, anxiety, and psychotic disorder patients. We conclude with two main observations related to the current state of the field. First, we found limited research in the form of published papers on this topic, perhaps due to the novelty of the topic and approach. While there are a larger number of studies reporting smartphone-based sleep monitoring in general patient populations, there are very few studies of digital sleep monitoring and intervention among individuals diagnosed with psychiatric disorders. Given the strong link between sleep quality and mental health symptoms, this is an important area for future investigation. Second, as this field continues to advance rapidly, we anticipate that many more studies in the future will make use of this approach. It is likely that there are currently many more studies in progress that were not captured in our review. Importantly, while most of the studies in our review were pilot studies, the findings highlight the feasibility and acceptability of using smartphones for subjective assessment and objective monitoring of sleep among individuals with depression, anxiety, and psychotic disorders. At present, smartphone technologies do not appear to offer the quality or depth of sleep data compared to PSG, though it is plausible that as the technology and methods improve, new possibilities for detecting sleep problems in individuals with psychiatric disorders will emerge. Smartphones are ubiquitous and they offer a simple and practical tool that may offer clinical value and utility. The early findings summarized here are promising and emphasize this as an important area worthy of further exploration.

## References

[1] • Bhugra D, Tasman A, Pathare S, Priebe S, Smith S, Torous J, Arbuckle MR, Langford A, Alarcón RD, Chiu HF, First MB. The WPA-lancet psychiatry commission on the future of psychiatry. The Lancet Psychiatry. 2017;4(10):775-818. **This article describes several key priorities for psychiatry over the next decade and in particular focuses on the role of digital technologies, as well as the potential challenges and promising opportunities.**

[2] Kalucy Megan J., Grunstein Ron, Lambert Timothy, Glozier Nicholas (2013). Obstructive sleep apnoea and schizophrenia – A research agenda. Sleep Medicine Reviews.

[3] Monti Jaime M., BaHammam Ahmed S., Pandi-Perumal Seithikurippu R., Bromundt Vivien, Spence D. Warren, Cardinali Daniel P., Brown Gregory M. (2013). Sleep and circadian rhythm dysregulation in schizophrenia. Progress in Neuro-Psychopharmacology and Biological Psychiatry.

[4] Benca Ruth M., Okawa Masako, Uchiyama Makoto, Ozaki Shigeru, Nakajima Toru, Shibui Kayo, Obermeyer William H. (1997). Sleep and mood disorders. Sleep Medicine Reviews.

[5] Malik S, Kanwar A, Sim LA, Prokop LJ, Wang Z, Benkhadra K, Murad MH. The association between sleep disturbances and suicidal behaviors in patients with psychiatric diagnoses: a systematic review and meta-analysis. Systematic reviews. 2014;3(1):18. 10.1186/2046-4053-3-18 PMC3945796 24568642

[6] Neckelmann D, Mykletun A, Dahl A (2007). Chronic insomnia as a risk factor for developing anxiety and depression. Sleep.

[7] Franzen PL, Buysse DJ (2008). Sleep disturbances and depression: risk relationships for subsequent depression and therapeutic implications.

[8] Cohrs Stefan (2008). Sleep Disturbances in Patients with Schizophrenia. CNS Drugs.

[9] Keshavan MS, Diwadkar V, Montrose DM, Stanley J, Pettegrew JW (2004). Premorbid characterization in schizophrenia: the Pittsburgh High Risk Study. World Psychiatry. World Psychiatry.

[10] Benson KL (2006). Sleep in schizophrenia: impairments, correlates, and treatment. Psychiatr Clin North Am.

[11] Myles H, Myles N, Antic NA, Adams R, Chandratilleke M, Liu D, Galletly C (2016). Obstructive sleep apnea and schizophrenia: a systematic review to inform clinical practice. Schizophr Res.

[12] Ohayon Maurice M. (2003). The Effects of Breathing-Related Sleep Disorders on Mood Disturbances in the General Population. The Journal of Clinical Psychiatry.

[13] Chouinard S., Poulin J., Stip E., Godbout R. (2004). Sleep in Untreated Patients With Schizophrenia: A Meta-Analysis. Schizophrenia Bulletin.

[14] Hoffmann R, Hendrickse W, Rush AJ, Armitage R (2000). Slow-wave activity during non-REM sleep in men with schizophrenia and major depressive disorders. Psychiatry Res.

[15] Keshavan MS, Reynolds CF, Kupfer DJ (1990). Electroencephalographic sleep in schizophrenia: a critical review. Compr Psychiatry.

[16] Harvey AG, Talbot LS, Gershon A (2009). Sleep disturbance in bipolar disorder across the lifespan. Clin Psychol (New York).

[17] Keshavan MS, Montrose DM, Miewald JM, Jindal RD (2011). Sleep correlates of cognition in early course psychotic disorders. Schizophr Res.

[18] Manoach DS, Demanuele C, Wamsley EJ, Vangel M, Montrose DM, Miewald J, Keshavan MS (2014). Sleep spindle deficits in antipsychotic-naïve early course schizophrenia and in non-psychotic first-degree relatives. Front Hum Neurosci.

[19] Wamsley EJ, Tucker MA, Shinn AK, Ono KE, McKinley SK, Ely AV, Manoach DS (2012). Reduced sleep spindles and spindle coherence in schizophrenia: mechanisms of impaired memory consolidation?. Biol Psychiatry.

[20] Zanini MA, Castro J, Cunha GR, Asevedo E, Pan PM, Bittencourt L, Brietzke E (2015). Abnormalities in sleep patterns in individuals at risk for psychosis and bipolar disorder. Schizophr Res.

[21] Lopez J, Hoffmann R, Armitage R (2010). Reduced sleep spindle activity in early-onset and elevated risk for depression. J Am Acad Child Adolesc Psychiatry.

[22] Baglioni Chiara, Nanovska Svetoslava, Regen Wolfram, Spiegelhalder Kai, Feige Bernd, Nissen Christoph, Reynolds Charles F., Riemann Dieter (2016). Sleep and mental disorders: A meta-analysis of polysomnographic research. Psychological Bulletin.

[23] Ben-Zeev Dror, Scherer Emily A., Wang Rui, Xie Haiyi, Campbell Andrew T. (2015). Next-generation psychiatric assessment: Using smartphone sensors to monitor behavior and mental health. Psychiatric Rehabilitation Journal.

[24] Gloster AT, Richard D, Himle J, Koch E, Anson H, Lokers L, Thornton J (2008). Accuracy of retrospective memory and covariation estimation in patients with obsessive-compulsive disorder. Behav Res Ther.

[25] Dagan Yaron, Zinger Yaffa, Lavie Peretz (1997). Actigraphic sleep monitoring in posttraumatic stress disorder (PTSD) patients. Journal of Psychosomatic Research.

[26] Ancoli-Israel S, Cole R, Alessi C, Chambers M, Moorcroft W, Pollak CP (2003). The role of actigraphy in the study of sleep and circadian rhythms. Sleep.

[27] Ancoli-Israel Sonia, Martin Jennifer L., Blackwell Terri, Buenaver Luis, Liu Lianqi, Meltzer Lisa J., Sadeh Avi, Spira Adam P., Taylor Daniel J. (2015). The SBSM Guide to Actigraphy Monitoring: Clinical and Research Applications. Behavioral Sleep Medicine.

[28] Sadeh A, Hauri PJ, Kripke DF, Lavie P (1995). The role of actigraphy in the evaluation of sleep disorders. Sleep.

[29] Gershon A, Thompson WK, Eidelman P, McGlinchey EL, Kaplan KA, Harvey AG (2012). Restless pillow, ruffled mind: sleep and affect coupling in interepisode bipolar disorder. J Abnorm Psychol.

[30] Kaplan Katherine A, Talbot Lisa S, Gruber June, Harvey Allison G (2012). Evaluating sleep in bipolar disorder: comparison between actigraphy, polysomnography, and sleep diary. Bipolar Disorders.

[31] Korszun A, Young EA, Engleberg NC, Brucksch CB, Greden JF, Crofford LA (2002). Use of actigraphy for monitoring sleep and activity levels in patients with fibromyalgia and depression. J Psychosom Res.

[32] MCCALL CATHERINE, MCCALL W. VAUGHN (2011). Comparison of actigraphy with polysomnography and sleep logs in depressed insomniacs. Journal of Sleep Research.

[33] Prociow Pawel, Crowe John (2011). Personalised ambient monitoring for people with bipolar disorder. International Clinical Psychopharmacology.

[34] Robillard R, Hermens DF, Naismith SL, White D, Rogers NL, Ip TK, Mullin SJ, Alvares GA, Guastella AJ, Smith KL, Rong Y. Ambulatory sleep-wake patterns and variability in young people with emerging mental disorders. Journal of psychiatry & neuroscience: JPN. 2015;40(1):28. 10.1503/jpn.130247 PMC4275328 25203899

[35] Berle JO, Hauge ER, Oedegaard KJ, Holsten F, Fasmer OB. Actigraphic registration of motor activity reveals a more structured behavioural pattern in schizophrenia than in major depression. BMC research notes. 2010;3(1):149. 10.1186/1756-0500-3-149 PMC2890507 20507606

[36] Emens Jonathan, Lewy Alfred, Kinzie John Mark, Arntz Diana, Rough Jennifer (2009). Circadian misalignment in major depressive disorder. Psychiatry Research.

[37] Jean–Louis Girardin, Mendlowicz Mauro V, Gillin J.Christian, Rapaport Mark H, Kelsoe John R, Zizi Ferdinand, Landolt Hans-Peter, von Gizycki Hans (2000). Sleep estimation from wrist activity in patients with major depression. Physiology & Behavior.

[38] Abdullah Saeed, Choudhury Tanzeem (2018). Sensing Technologies for Monitoring Serious Mental Illnesses. IEEE MultiMedia.

[39] Torous John, Keshavan Matcheri (2018). A new window into psychosis: The rise digital phenotyping, smartphone assessment, and mobile monitoring. Schizophrenia Research.

[40] Center PR. The smartphone difference. Pew Research Center. 2015:1-60.

[41] Saeb Sohrab, Cybulski Thaddeus R, Kording Konrad P, Mohr David C (2017). Scalable Passive Sleep Monitoring Using Mobile Phones: Opportunities and Obstacles. Journal of Medical Internet Research.

[42] Abdullah S, Matthews M, Murnane EL, Gay G, Choudhury T. Towards circadian computing: early to bed and early to rise makes some of us unhealthy and sleep deprived. InProceedings of the 2014 ACM international joint conference on pervasive and ubiquitous computing 2014;673-684. ACM.

[43] Murnane EL, Abdullah S, Matthews M, Choudhury T, Gay G. Social (media) jet lag: How usage of social technology can modulate and reflect circadian rhythms. InProceedings of the 2015 ACM International Joint Conference on Pervasive and Ubiquitous Computing 2015;843-854. ACM.

[44] • Aledavood T, Lehmann S, Saramäki J. Social network differences of chronotypes identified from mobile phone data. EPJ Data Science. 2018;7(1):46. **This study shows that sleep behaviors of students have an impact on the structure of their social networks and their social interaction. Those who stay up late and wake up late (owls) are more central in the social network of students than those who go to sleep early and wake up early (larks).**

[45] Aledavood T, Lehmann S, Saramäki J. Digital daily cycles of individuals. 3(2015):73.

[46] •• Staples P, Torous J, Barnett I, Carlson K, Sandoval L, Keshavan M, Onnela JP. A comparison of passive and active estimates of sleep in a cohort with schizophrenia. NPJ schizophrenia. 2017;3(1):37. **This article reports on a study demonstrating that smarphones show feasibility for monitoring sleep among persons with schizophrenia and may assist in the identification of sleep abnormalities in this patient group. For example, phone-based accelerometer data was used to infer sleep duration and showed moderate correlations with self-assessment of sleep duration.** 10.1038/s41537-017-0038-0 PMC5643440 29038553

[47] Zulueta J, Piscitello A, Rasic M, Easter R, Babu P, Langenecker SA, Leow A (2018). Predicting mood disturbance severity with mobile phone keystroke metadata: a biaffect digital phenotyping study. J Med Internet.

[48] Babson Kimberly A, Ramo Danielle E, Baldini Lisa, Vandrey Ryan, Bonn-Miller Marcel O (2015). Mobile App-Delivered Cognitive Behavioral Therapy for Insomnia: Feasibility and Initial Efficacy Among Veterans With Cannabis Use Disorders. JMIR Research Protocols.

[49] Bauer Michael, Grof Paul, Gyulai Laszlo, Rasgon Natalie, Glenn Tasha, Whybrow Peter C (2004). Using technology to improve longitudinal studies: self-reporting with ChronoRecord in bipolar disorder. Bipolar Disorders.

[50] Depp Colin A., Mausbach Brent, Granholm Eric, Cardenas Veronica, Ben-Zeev Dror, Patterson Thomas L., Lebowitz Barry D., Jeste Dilip V. (2010). Mobile Interventions for Severe Mental Illness. The Journal of Nervous and Mental Disease.

[51] Faurholt-Jepsen Maria, Vinberg Maj, Frost Mads, Christensen Ellen Margrethe, Bardram Jakob E, Kessing Lars Vedel (2015). Smartphone data as an electronic biomarker of illness activity in bipolar disorder. Bipolar Disorders.

[52] Lemola Sakari, Perkinson-Gloor Nadine, Brand Serge, Dewald-Kaufmann Julia F., Grob Alexander (2014). Adolescents’ Electronic Media Use at Night, Sleep Disturbance, and Depressive Symptoms in the Smartphone Age. Journal of Youth and Adolescence.

[53] Schaffer Ayal, Kreindler David, Reis Catherine, Levitt Anthony J. (2013). Use of Mental Health Telemetry to Enhance Identification and Predictive Value of Early Changes During Augmentation Treatment of Major Depression. Journal of Clinical Psychopharmacology.

[54] Kane John M., Perlis Roy H., DiCarlo Lorenzo A., Au-Yeung Kityee, Duong Jessie, Petrides Georgios (2013). First Experience With a Wireless System Incorporating Physiologic Assessments and Direct Confirmation of Digital Tablet Ingestions in Ambulatory Patients With Schizophrenia or Bipolar Disorder. The Journal of Clinical Psychiatry.

[55] Ben-Zeev D, Brenner CJ, Begale M, Duffecy J, Mohr DC, Mueser KT. Feasibility, acceptability, and preliminary efficacy of a smartphone intervention for schizophrenia. 2014;40(6):1244–53. 10.1093/schbul/sbu033 PMC4193714 24609454

[56] Ben-Zeev Dror, Brian Rachel M., Aschbrenner Kelly A., Jonathan Geneva, Steingard Sandra (2018). Video-based mobile health interventions for people with schizophrenia: Bringing the “pocket therapist” to life. Psychiatric Rehabilitation Journal.

[57] Matthews Mark, Abdullah Saeed, Murnane Elizabeth, Voida Stephen, Choudhury Tanzeem, Gay Geri, Frank Ellen (2016). Development and Evaluation of a Smartphone-Based Measure of Social Rhythms for Bipolar Disorder. Assessment.

[58] Abdullah Saeed, Matthews Mark, Frank Ellen, Doherty Gavin, Gay Geri, Choudhury Tanzeem (2016). Automatic detection of social rhythms in bipolar disorder. Journal of the American Medical Informatics Association.

[59] Faurholt-Jepsen Maria, Frost Mads, Vinberg Maj, Christensen Ellen Margrethe, Bardram Jakob E., Kessing Lars Vedel (2014). Smartphone data as objective measures of bipolar disorder symptoms. Psychiatry Research.

[60] Ben-Zeev Dror, Brian Rachel, Wang Rui, Wang Weichen, Campbell Andrew T., Aung Min S. H., Merrill Michael, Tseng Vincent W. S., Choudhury Tanzeem, Hauser Marta, Kane John M., Scherer Emily A. (2017). CrossCheck: Integrating self-report, behavioral sensing, and smartphone use to identify digital indicators of psychotic relapse. Psychiatric Rehabilitation Journal.

[61] Wang R, Wang W, Aung MS, Ben-Zeev D, Brian R, Campbell AT, Walsh M (2017). Predicting symptom trajectories of schizophrenia using mobile sensing. Conference Proceedings of the ACM on Interactive, Mobile, Wearable and Ubiquitous Technologies.

[62] Meyer Nicholas, Kerz Maximilian, Folarin Amos, Joyce Dan W, Jackson Richard, Karr Chris, Dobson Richard, MacCabe James (2018). Capturing Rest-Activity Profiles in Schizophrenia Using Wearable and Mobile Technologies: Development, Implementation, Feasibility, and Acceptability of a Remote Monitoring Platform. JMIR mHealth and uHealth.

[63] Al-Mardini Mamoun, Aloul Fadi, Sagahyroon Assim, Al-Husseini Luai (2014). Classifying obstructive sleep apnea using smartphones. Journal of Biomedical Informatics.

[64] Al-Mardini M, Aloul F, Sagahyroon A, Al-Husseini L. On the use of smartphones for detecting obstructive sleep apnea. In13th IEEE International Conference on BioInformatics and BioEngineering 2013;1-4. IEEE.

[65] Behar Joachim, Roebuck Aoife, Domingos João S, Gederi Elnaz, Clifford Gari D (2013). A review of current sleep screening applications for smartphones. Physiological Measurement.

[66] Walch Olivia J., Cochran Amy, Forger Daniel B. (2016). A global quantification of “normal” sleep schedules using smartphone data. Science Advances.

[67] Cuttone Andrea, Bækgaard Per, Sekara Vedran, Jonsson Håkan, Larsen Jakob Eg, Lehmann Sune (2017). SensibleSleep: A Bayesian Model for Learning Sleep Patterns from Smartphone Events. PLOS ONE.

[68] Behar Joachim, Roebuck Aoife, Shahid Mohammed, Daly Jonathan, Hallack Andre, Palmius Niclas, Stradling John, Clifford Gari D. (2015). SleepAp: An Automated Obstructive Sleep Apnoea Screening Application for Smartphones. IEEE Journal of Biomedical and Health Informatics.

[69] Garde A, Dehkordi P, Wensley D, Ansermino JM, Dumont GA. Pulse oximetry recorded from the Phone Oximeter for detection of obstructive sleep apnea events with and without oxygen desaturation in children. In2015 37th Annual International Conference of the IEEE Engineering in Medicine and Biology Society (EMBC) 2015;7692-7695. IEEE. 10.1109/EMBC.2015.7320174 26738074

[70] Tal Asher, Shinar Zvika, Shaki David, Codish Shlomi, Goldbart Aviv (2017). Validation of Contact-Free Sleep Monitoring Device with Comparison to Polysomnography. Journal of Clinical Sleep Medicine.

[71] Sterr A, Ebajemito JK, Mikkelsen KB, Bonmati-Carrion MA, Santhi N, Della Monica C, Grainger L, Atzori G, Revell V, Debener S, Dijk DJ. Sleep EEG Derived From Behind-the-Ear Electrodes (cEEGrid) Compared to Standard Polysomnography: A Proof of Concept Study. Frontiers in human neuroscience. 2018;12. 10.3389/fnhum.2018.00452 PMC6276915 30534063

[72] Aledavood Talayeh, Triana Hoyos Ana Maria, Alakörkkö Tuomas, Kaski Kimmo, Saramäki Jari, Isometsä Erkki, Darst Richard K (2017). Data Collection for Mental Health Studies Through Digital Platforms: Requirements and Design of a Prototype. JMIR Research Protocols.

[73] Bhat S, Ferraris A, Gupta D, Mozafarian M, DeBari VA, Gushway-Henry N, Gowda SP, Polos PG, Rubinstein M, Seidu H, Chokroverty S. Is there a clinical role for smartphone sleep apps? Comparison of sleep cycle detection by a smartphone application to polysomnography. Journal of Clinical Sleep Medicine. 2015;11(07):709-15. 10.5664/jcsm.4840 PMC4481053 25766719

[74] •• Kolla BP, Mansukhani S, Mansukhani MP. Consumer sleep tracking devices: a review of mechanisms, validity and utility. Expert review of medical devices. 2016;13(5):497-506. **This review evaluates the literature reporting the accuracy of consumer sleep tracking devices compared to more conventional sleep measurement methods. Importantly, this review highlights that among normal subjects, consumer tracking devices tend to underestimate sleep disruptions and overestimate total sleep times and sleep efficiency.** 10.1586/17434440.2016.1171708 27043070

[75] Yu Jessica S, Kuhn Eric, Miller Katherine E, Taylor Katherine (2018). Smartphone apps for insomnia: examining existing apps’ usability and adherence to evidence-based principles for insomnia management. Translational Behavioral Medicine.

[76] Espie Colin A., Emsley Richard, Kyle Simon D., Gordon Christopher, Drake Christopher L., Siriwardena A. Niroshan, Cape John, Ong Jason C., Sheaves Bryony, Foster Russell, Freeman Daniel, Costa-Font Joan, Marsden Antonia, Luik Annemarie I. (2019). Effect of Digital Cognitive Behavioral Therapy for Insomnia on Health, Psychological Well-being, and Sleep-Related Quality of Life: A Randomized Clinical Trial. JAMA Psychiatry.

